# Learning to walk before we run: what can medical education learn from the human body about integrated care

**DOI:** 10.5334/ijic.1128

**Published:** 2013-05-24

**Authors:** Eron G Manusov, Daniel P Marlowe, Deborah J Teasley

**Affiliations:** Duke Southern Regional Area Health Education (SRAHEC), Family Medicine Residency, Fayetteville, NC, USA; Duke Southern Regional Area Health Education (SRAHEC), Family Medicine Residency, Fayetteville, NC, USA; Duke Southern Regional Area Health Education (SRAHEC), Family Medicine Residency, Fayetteville, NC, USA

**Keywords:** integrated care, medical education, residency

## Abstract

True integration requires a shift in all levels of medical and allied health education; one that emphasizes team learning, practicing, and evaluating from the beginning of each students’ educational experience whether that is as physician, nurse, psychologist, or any other health profession. Integration of healthcare services will not occur until medical education focuses, like the human body, on each system working inter-dependently and cohesively to maintain balance through continual change and adaptation. The human body develops and maintains homeostasis by a process of communication: true integrated care relies on learned interprofessionality and ensures shared responsibility and practice.

## Learning to walk before we run: what can medical education learn from the human body about integrated care?

Over the last 18 months, the Duke/SRAHEC Family Medicine Residency in Fayetteville, North Carolina, USA has shifted to make integrated care a core tenant of our practice and educational models. A transition of this magnitude was not made in isolation, but rather, resulted as a culmination of several different projects coalescing around the fundamental belief that primary healthcare is best delivered in interdisciplinary collaborative teams. However, as we attempted to change our practice structure through incorporating multiple discipline consultations in our outpatient clinic, we realized that this transition was incomplete without a concurrent emphasis on teaching our family medicine residents about integrated care specifically. Supported by a Kate B. Reynolds Foundation grant, we subsequently developed an Integrated Care Curriculum that added a structured process for teaching integrated care theory, clinical practice, and practice management in graduate medical education. The curriculum development mirrored the process of integration since it was collaborative, organic; based on the needs of the organization as seen by its collective members, and constructed with outcomes in mind. This educational experience promoted integration across curriculums, rotations, organizational structures, practice structures, and community stakeholders.

Ultimately, this organizational shift required three interconnected elements: (i) finding the best time/place to begin the change process, (ii) adopting a common language and metaphor to conceptualize that process, and (iii) developing practice structures that promoted function as an integrated unit. While our answer to the final element was the creation of a practice model and curriculum whose core axiom was ‘we plan, execute, and evaluate together,’ the answers to the other two elements were slightly more elusive. We concluded that even with our graduate education curriculum and practice model we were still approaching physicians far too late in their education. True integration, we posit, requires a shift in all levels of medical and allied health education; one that emphasizes team learning, practicing, and evaluating from the very beginning of each students’ educational experience whether that is as physician, nurse, psychologist, or any other health profession. As we discussed, our realization that true integration must begin with how health professionals are trained, we also concluded that how better to talk about that process than through using the best example of systemic integration—the human body. When thinking about the body, although one might point to the heart or brain as the ‘most’ important organ, the reality remains that every cell and organ can only function appropriately in relationship to whole. This serves as the perfect metaphor regarding how we see integration at both conceptual and practice levels.

The increased complexity and unsustainable cost of healthcare in the US mandates a shift to team-based integration and care delivery, however, poorly integrated care can negatively affect healthcare cost and patient outcomes [1]. Delivery of quality healthcare requires significant coordination across disciplines and settings. Healthcare education is as fragmented as healthcare delivery; each discipline learning, practicing and evaluating independently. In the US, the Patient Centered Medical Home (PCMH) is the practice model that showcases healthcare integration, but in order to meet the model’s goals, significant transformation to inter-professional education is also required, a process that is incongruent with the current American medical education system. Our theory is that like any socialization process, the difficulty for health professionals to truly provide integrated medical care begins very early in their education and is reinforced by societal emphasis on specialization. Healthcare education from the university to the post-graduate level is often co-located on campus, but each discipline learns separately. Medical schools in the US are within throwing distance from colleges of nursing, social work, and psychology, yet often minimally interact. This process rarely emphasizes integration of planning, researching, thinking, or practicing as teams. In fact, medical students are trained to be the ‘captain of the ship’ where the final decisions and responsibilities lie with them as the physician. When physicians are trained and society reinforces the physician as the acknowledged director and leader, it is no wonder that collaboration and integration is a difficult process.

This educational separation is implicit within medical training curricula, which the social scientist, Barret Michalec, refers to as the ‘hidden curriculum,’ and is evidenced by the cognitive and emotional distancing from non-medical students by medical students. Medical students look at other healthcare providers such as the nursing students, social work students, and pharmacy students as contributing only a small portion to their entire curriculum and overall educational experience. However, this is not only endemic in academic medicine, but is a trans-disciplinary process equated with students (regardless of discipline) developing a distinct professional identity [2]. Although in the US we now emphasize inter-professional healthcare at the level of practice, the continued explicit and implicit support of the rigid separation of disciplines at the educational level results in a process that yields at best collaboration, and at worst discontent, animosity, fragment learning, fragmented practice and subsequently fragmented care.

Interprofessionality, a word coined by D’Amour and colleagues, is the process by which professionals reflect upon and develop ways of practicing that provides an integrated and cohesive approach to the needs of the client-family population [2]. Integrated, inter-professional and team based care will occur when we conceptualize both our educational and practice structures as a cooperative collective. The metaphor that we offer is the human body that begins as two cells that divide repeatedly. Pleuripotent cells develop into specialized tissues in the milieu and influences of a complex system of hormones, electrical signals, and reactions. Not orchestrated by one system alone, the cells grow, change, and function according to the needs of the organism and the interdependency between the organism and its environment. Cells form, change, live, and die according to ‘rules, regulations, and accrediting bodies,’ and that without all parts of the system working together ‘inter-professionally’ the system would lapse into dysfunction.

Interprofessionality and the subsequent integration of healthcare services will not occur until medical education focuses, like the human body, on each system working inter-dependently and cohesively to maintain balance through continual change and adaptation—a process known as morphostasis. Learning and working in the same room allows us to be cordial and play nicely together. Incontrovertible patient centered, integrated and collaborative care, however, will not occur until all disciplines train, think, create, and seek solutions as a unit. This does not mean to do away with disciplinary specialization, but to engender education (at both undergraduate and graduate levels) to actualize integrated function and care. All healthcare educators need to plan, execute, and evaluate together to teach integrated care to all disciplines. Like the human body, each ‘cell’ may form new organs or specialized tissues, but these specializations always take place in the context of the entire body as a whole. Once medical education learns how to accomplish this task of shared learning, practice, and responsibility, integrated inter-professional collaboration will grow naturally from this educational process and lead to improved, cost effective, cohesive care and a truly patient centered care model.

## From the authors

Eron G. Manusov, MD, is the Vice President of Clinical Education and Services (CEAS) for the Southern Regional Area Health Education Center (SR-AHEC). CEAS comprises a marriage and family therapy residency, pharmacy doctoral residency, an allopathic and osteopathic family medicine residency and offers local, state and national integrated workshops. Dr. Manusov’s extensive academic career includes teaching at the Uniformed Services University School of Medicine and The Florida State University College of Medicine, funded research, and an extensive list of publications and national and international presentations. The three authors developed the only known specific longitudinal and developmental integrated care curriculum specific for graduate medical education.

Dan Marlowe, PhD, LMFT is the director of Applied Psychosocial Medicine for Southern Regional AHEC in Fayetteville, NC where he oversees integrated care education for family medicine, pharmacy and medical family therapy residents. Over the course of his career, Dr. Marlowe has helped develop and implement integrated/collaborative care programs at a federally qualified health center, patient centered medical home, family medicine residency program and major university cancer center. He has published both nationally and internationally on integrated/collaborative care, as well as presented at regional, state and national levels on integrated care program development, teaching integration in graduate medical education, and the financial stability of integrated primary care models.

Deborah Teasley holds a PhD in Health Administration from Texas A&M University and Bachelor’s and Master’s degrees in Nursing from The University of Texas Medical Branch in Galveston, TX. She is the President and Chief Executive Officer (CEO) of the Southern Regional Area Health Education Center in Fayetteville and adjunct Assistant Dean at Duke University Medical Center. She has extensive experience in health care management including CEO of an urban hospital and corporate Senior Vice President and Chief Operating Officer of a large healthcare system. As adjunct faculty, Dr. Teasley Teaches Health Policy and strategic management. Her special interests are in leadership and organizational development including appreciative inquiry. Dr. Teasley is a Fellow in the American College of Healthcare Executives and has served as a Regent for the College. She has published and presented in multiple forums at the national level.
